# Systematic underestimation of the epigenetic clock and age acceleration in older subjects

**DOI:** 10.1186/s13059-019-1810-4

**Published:** 2019-12-17

**Authors:** Louis Y. El Khoury, Tyler Gorrie-Stone, Melissa Smart, Amanda Hughes, Yanchun Bao, Alexandria Andrayas, Joe Burrage, Eilis Hannon, Meena Kumari, Jonathan Mill, Leonard C. Schalkwyk

**Affiliations:** 1 School of Life Sciences, University of Essex, Colchester, UK; 2 Present Address: Department of Molecular Pharmacology and Experimental Therapeutics, Mayo Clinic, Rochester, MN USA; 3 Institute for Social and Economic Research, University of Essex, Colchester, UK; 4 MRC Integrative Epidemiology Unit - University of Bristol, Bristol, UK; 5 Medical School, University of Exeter, Exeter, UK

**Keywords:** DNA methylation, Epigenetic clock, Age acceleration

## Abstract

**Background:**

The Horvath epigenetic clock is widely used. It predicts age quite well from 353 CpG sites in the DNA methylation profile in unknown samples and has been used to calculate “age acceleration” in various tissues and environments.

**Results:**

The model systematically underestimates age in tissues from older people. This is seen in all examined tissues but most strongly in the cerebellum and is consistently observed in multiple datasets. Age acceleration is thus age-dependent, and this can lead to spurious associations. The current literature includes examples of association tests with age acceleration calculated in a wide variety of ways.

**Conclusions:**

The concept of an epigenetic clock is compelling, but caution should be taken in interpreting associations with age acceleration. Association tests of age acceleration should include age as a covariate.

## Background

Subject age is a piece of data available in almost every study in which DNA methylation profiles are obtained. There is thus a huge amount of cross-sectional data in which it can be seen that the methylation level of many CpG sites varies with subject age [1–4], which, amongst other processes, could reflect developmental changes, cellular aging, cumulative environmental effects, and changes in cell-type composition. Exploring these sources of variation could give insights into age-related processes. Predicted ages can also provide a valuable quality control and identity check on data in EWAS studies [5–8].

Horvath [8] used a large collection (*n* > 8000) of publicly available Illumina HumanMethylation array data on multiple tissue types to train and test a model for age prediction from 353 CpG loci. This “epigenetic clock” continues to be widely used and is extremely valuable as a way of estimating ages of samples from unknown donors and possibly as an indicator of whether there are alterations in the aging rate of certain tissues or individuals. Although the epigenetic clock developed by Horvath [8] provides an estimate of age, the testing data used in generating this clock did not have a large representation of tissue from elderly individuals and as such it is unclear if the clock is accurate in older age groups, or those with age-related diseases.

We have previously published an epigenome-wide association study (EWAS) in Alzheimer disease (AD), utilizing four brain tissues and pre-mortem blood, and demonstrated DNA methylation differences at specific loci in a tissue-specific manner [9]. This dataset offers a good opportunity to examine the properties of the Horvath [8] clock on different tissues in both elderly non-demented individuals and AD sufferers. We further explore the properties of the model using a cross-sectional population sample from the UK Household Longitudinal study (UKHLS), which has a wide range of ages [10].

## Results

### Age estimation

**Fig. 1:**
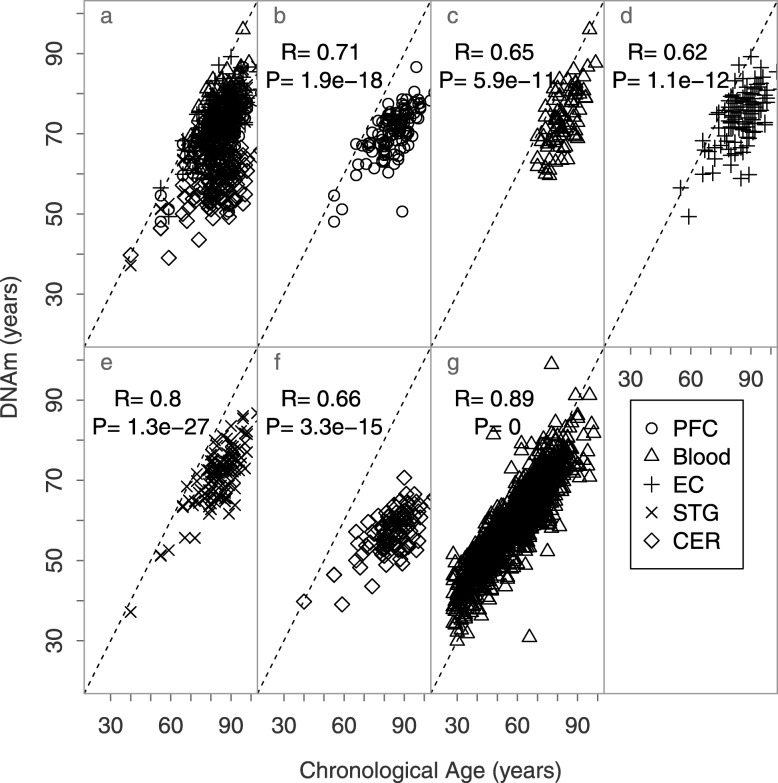
Scatterplots of chronological vs DNAm ages of brain and blood samples. Each point corresponds to an independent sample. The dotted line is the *y* = *x* bisector line, and the solid lines correspond to the regression line of each tissue. PFC, prefrontal cortex; EC, entorhinal cortex; STG, the superior temporal gyrus; CER, cerebellum (data from [9] for panels **a**–**f** and [10] for panel **g**)

Initially, we observed in our AD dataset [9] that ages were strikingly underestimated using the Horvath [8] clock. Indeed, in this elderly data set, across multiple brain regions and in blood, the model did predict age but with a slope of predicted against actual age clearly less than 1 (Fig. 1a–f). This was borne out in the much larger UKHLS set of blood DNA samples measured with the Illumina EPIC array [10] (Fig. 1g).

**Fig. 2:**
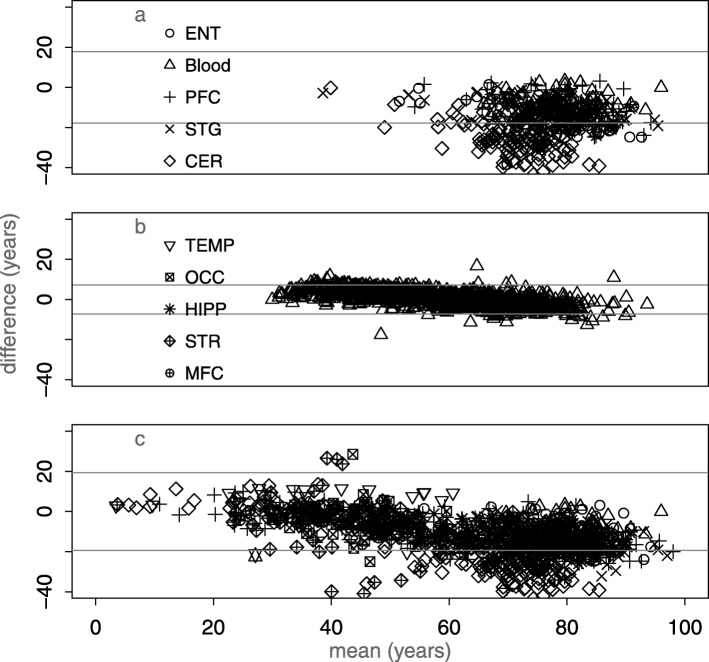
Mean-difference (Bland-Altman) plots showing the difference between Horvath model age and chronological age. **a** Elderly brain: AD data set [9], **b** population blood sample [10], and **c** additional publicly available datasets (see Additional file 1: Table S1). The horizontal lines in each case are at ± 1.96 * sd; for normally distributed difference due to error 5% of points would lie outside these and in each case many more do

The discrepancy can be more clearly demonstrated with a mean-difference (Bland-Altman) plot (thanks to a reviewer for this suggestion). There is a trend to larger discrepancy with age in the the AD data set, UKHLS, and a collection of additional datasets listed in Additional file 1: Table S1 (Fig. 2).

**Fig. 3:**
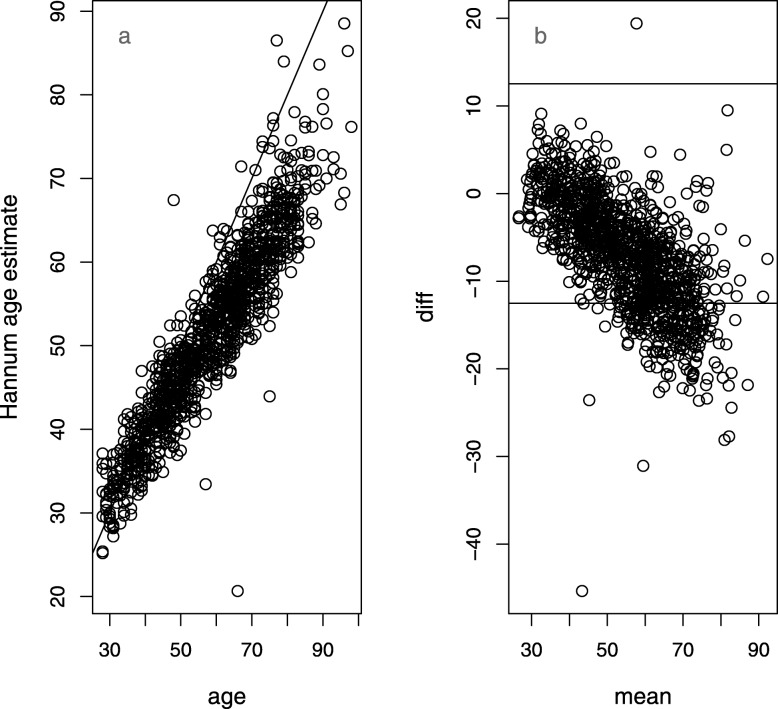
Plots showing the difference between Hannum model age and chronological age in the UKHLS [10] data set. **a** Scatter plot. **b** Mean-difference (Bland-Altman) plot

We focus in this study on the characteristics of the Horvath model [8] because it remains widely used and because it is designed to be applicable across tissues. For the UKHLS dataset, we also looked at the blood-specific Hannum [6] model. This model is simpler than the Horvath one, with 71 coefficients. On the UKHLS dataset, it shows a very similar deviation, with increasing underestimation in advanced age (Fig. 3). There is an overlap of 6 loci between the two models.

### Clock properties

**Fig. 4:**
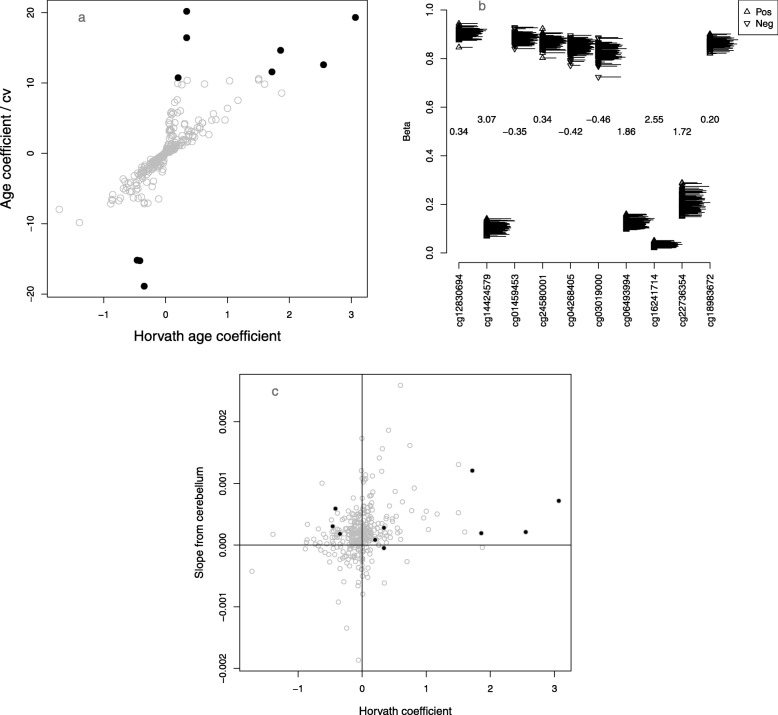
Exploration of model coefficients in the elderly cerebellum. **a** Scatter plot of age coefficients against their influence score (coefficient from Horvath [8]/coefficient of variation in our data). The 10 most influential loci (largest scores by absolute value) are plotted in black, **b** 10 most influential loci, with the ages represented as a rug on the right-hand side of each strip chart. The Horvath coefficients are shown in the center, and their sign is also denoted by the direction of the triangles, upward facing for positive and downward facing for negative. **c** Scatter plot of Horvath [8] coefficients against their linear-model age coefficients in our data. The 10 most influential probes are shown in black

Next, to get some insight into the behavior of the clock, we dissected possible reasons for its nonlinear response. The absolute values of the coefficients in the Horvath model range from 5.9e−6 to 3.07, so some of the CpG sites are much more important than others. To investigate this, we make an index of the influence of each locus on the age estimate by dividing the absolute value of the coefficient from the Horvath clock by an index of dispersion from our data, the coefficient of variation. The ten highest ranked probes by this measure (Fig. 4a, black circles at the top and bottom of the plot) include examples of both small variance (and large coefficient) and large variance (and possibly smaller coefficient), although clearly the smallest coefficients are not going to make an appreciable difference to the age estimate no matter what the variance. Two of the ten most influential probes, cg22736354 and cg06493994, are also used in the Hannum model.

One simple way the clock rate could be reduced in later life is by saturation, i.e., the CpG sites used in the clock reaching either full methylation or complete demethylation. To investigate this, we dissect the ten most influential further (Fig. 4b). Of these ten probes, three (cg12830694, cg24580001, and cg02580606) might be candidates for saturation because they are highly methylated and expected from the model to be increasing with age.

To test this further, we fitted a regression line between chronological age and the beta values of each of the 353 loci and plotted the slopes against the Horvath coefficients. Of the ten most influential loci, four have a slope opposite in sign to the Horvath coefficient (cg08090772, cg03019000, cg04268405, and cg24580001) (Fig. 4c). Loci with the largest coefficients demonstrate the same direction of effect in our data as in the Horvath model. For this tissue and age range, many of the smaller coefficients are effectively random, but they may be influential in the model’s performance in other tissues. Slieker et al. [11] have shown that the majority of age effects are tissue-specific.

### Age acceleration associations

As an example of an association test using age acceleration, we examined whether age acceleration (calculated as the difference between DNAm age and chronological age) associates with AD neuropathology (measured using Braak score) in the London cohort [9]. We observed a weak association in some brain tissues. However, when age is included as a covariate, the association between age acceleration and AD pathology disappears. We also see this in the Mount Sinai cohort [12, 13] where no correlation was found between age acceleration and amyloid plaque levels when age is included as a covariate (Table 1).

**Table 1:** Regression analysis of epigenetic age acceleration of four brain tissues and blood from the London Brain Bank cohort [9] versus brain Braak stage and of two brain tissues from the Mount Sinai cohort [12] versus amyloid plaque levels

	London cohort		London cohort (age in model)		Mount Sinai cohort (age in model)	
	Coefficient	*P* value	Coefficient	*P* value	Coefficient	*P* value
Prefrontal cortex	0.434	0.164	− 0.345	0.107	− 0.029	0.629
Entorhinal cortex	0.969	0.004	0.424	0.125	–	–
Sup. Temporal Gyrus	0.653	0.019	− 0.062	0.786	0.004	0.944
Cerebellum	1.127	0.002	− 0.059	0.788	–	–
Blood	− 0.278	0.632	− 0.090	0.870	–	–

Shown for each cohort the coefficient and *P* value for the regression analysis between age acceleration and Braak stages (London cohort) or amyloid plaque levels (Mount Sinai cohort) with chronological age as a covariate

In a broad but non-comprehensive survey of the literature (Table 2), we observe a variety of methods of calculating age acceleration, and many studies do not correct for chronological age. Initially, Δ-age (the difference between chronological age and the DNAm predicted age) was reported, but alternative methods have since arisen: (1) the residual of regressing DNAm predicted age on chronological age (possibly in a model including covariates), (2) AgeAccel (difference between DNAm age value and the value predicted by a regression model in the control group), and (3) intrinsic (IEAA) and (4) extrinsic epigenetic age acceleration (EEAA) methods. Both IEAA and EEAA are methods applicable only on blood since they subtract out the effect of blood cell count [16, 18].

**Table 2:** Literature survey of age acceleration

Reference	Phenotype	AIM	SIG	Age acceleration method		
Horvath [14]	Obesity	No	Yes	Residuals of DNAm age regressed on chronological age		
Marioni et al. [15]	All-cause mortality	Yes	Yes	Δ-age		
Levine et al. [16]	Lung cancer incidence	Yes	Yes	IEAA		
Levine et al. [17]	Neuritic plaque	Yes	Yes	Residuals of DNAm age regressed on chronological age and sex		
Horvath et al. [18]	Parkinson’s disease	Yes	No	AgeAccel		
		Yes	No	IEAA		
		Yes	Yes	EEAA		
Horvath et al. [19]	Down syndrome	No	Yes	Residuals DNAm age regressed on chronological age in controls		
Marioni et al. [20]	Fitness variables	Yes	Yes	Residuals DNAm age regressed on chronological age		
Horvath et al. [21]	HIV	No	Yes	AgeAccel		
Horvath et al. [22]	Alzheimer in the cerebellum	Yes	No	Residual DNAm age against chronological age in non-cerebellar brain sample		
Horvath et al. [23]	Longevity	No	Yes	AgeAccel		
		No	Yes	IEAA		
		No	Yes	EEAA		
Walker [24]	Multifocal developmental disorders	No	No	Residuals DNAm age regressed on chronological age		
Lin et al. [25]	Life expectancy	Yes	Yes	Δ-age		
Perna [26]	All-cause mortality	Yes	Yes	Δ-age		
	Cancer mortality	Yes	Yes	Δ-age		
	Cardiovascular mortality	Yes	Yes	Δ-age		
Horvath [27]	Huntington’s disease	No	Yes	Residuals DNAm age regressed on chronological age		
Levine et al. [28]	Age at menopause	Yes	Yes	AgeAccel		
Horvath [29]	Race/ethnicity	Yes	Yes	EEAA		
Chen et al. [30]	Time of death	Yes	Yes	EEAA		
		Yes	Yes	IEAA		
		Yes	Yes	AgeAccel		
Simpkin et al. [31]	Birth weight	No	Yes	Δ-age		
Gao et al. [32]	Smoking status	Yes	No	Residuals DNAm age regressed on chronological age		
	Cumulative exposure	Yes	No	Residuals DNAm age regressed on chronological age		
	Cessation time	Yes	No	Residuals DNAm age regressed on chronological age		
Breitling et al. [33]	Frailty	Yes	Yes	Δ-age		
Ward-Caviness et al. [34]	Air pollution	No	Yes	Residuals DNAm age regressed on chronological age		
		Yes	Yes	EEAA		
		Yes	Yes	IEAA		
Levine et al. [35]	HIV-associated neurocognitive disorders	Yes	Yes	Residuals DNAm age regressed on chronological age		
Armstrong et al. [36]	Longevity	No	Yes	AgeAccel - Hannum		
McKinney et al. [37]	Schizophrenia duration	No	No	Residuals DNAm age regressed on chronological age		
Wolf et al. [38]	PTSD hyperarousal	No	Yes	Residuals DNAm age regressed on chronological age		
	PTSD severity	No	No	Residuals DNAm age regressed on chronological age		
Quach et al. [39]	Diet and lifestyle	Yes	Yes	EEAA		
	Diet and lifestyle	Yes	Yes	IEAA		
Binder et al. [40]	Time to menarche	No	Yes	AgeAccel		
	Pubertal tempo	No	Yes	AgeAccel		
	Breast fibro-glandular volume	No	Yes	AgeAccel		
Dugué [41]	Mortality	Yes	Yes	Residuals DNAm age regressed on chronological age		
		Yes	Yes	IEAA		

## Discussion

The Horvath epigenetic clock [8] has been of practical use in predicting the age of unknown samples and as a quality check in epigenetic research. Additional widely used age predictors specific for blood were published by Hannum [6] and Levine [42] (phenotype-based). Here we analyze the Horvath model, but the methods and many of the conclusions may be more widely applicable, in particular the Hannum clock model shows a similar underestimation of ages in elderly subjects.

The mechanism or mechanisms of the apparent change of gears in a person’s sixties are not clear. At least part of the effect with these models seems to be saturation, i.e., loci approaching the limits of 0 or 100% methylation. Another intriguing part of the picture, at least for the brain, could be 5-hydroxymethyl cytosine, which is present at appreciable levels in brain tissues, especially cerebellum which is characterized by elevated levels of 5-hydroxymethylcytosine (5hmC) [15]. We found that 31 out of the 353 Horvath clock sites were amongst the 65,663 elevated 5hmC probes found in the cerebellum by Lunnon et al. [15]. Of these, two sites (cg04268405, and cg24580001) are amongst the most influential sites (Fig. 4). Given that 5hmC is not distinguished from 5mC following bisulfite conversion, it is possible that age-associated changes to the 31 5hmC sites of the Horvath algorithm are offsetting the age predictions.

These two models both use a small fraction of the available age indexing GpGs, especially since much more comprehensive arrays are now in use, and in fact their site contents overlap. Although improved age prediction can no doubt be achieved by making use of additional informative loci, especially in tissue-specific models, we believe it is more important to use existing models with an awareness of their properties and limitations and not as a black box.

In addition to age prediction, the Horvath [8] paper also featured the idea of “age acceleration” in which discrepancies between DNA methylation (DNAm) age and chronological age might tell us something about the biological aging status of the organism. A number of positive association findings with age association, particularly mortality [43], make it compelling to think of the epigenetic clock as an index of an underlying aging program that adapts to health and environment. In light of the methodological variety though, we are concerned that the different epigenetic clocks, and the variety of age acceleration methods to choose from, lay a trap of potentially hidden multiple testing, as the temptation will be to survey the available methods for interesting results.

When comparing DNA methylation profiles across tissues, individuals, and other variables such as health, the dominant source of variation is the tissue, or more precisely the cell type. It is reasonable to suppose that this developmental blueprint can change over time in response to the environment, or simply drift or decay. This point of view corresponds roughly with the “epigenetic maintenance” model posited by Horvath [8], and developed further by Horvath and Raj [44].

The “decay clock” or epigenetic maintenance models are perhaps more likely to be accurate than a biochemical aging clock, but they are somewhat at odds with the age acceleration concept. Association tests with age acceleration are very common but should be treated with caution, especially if the effect is small. As shown in this study, in the latter third of the human age range, where such associations are most likely observed, negative age acceleration increases with age. This means that any phenotype associated with age will appear to be associated with age acceleration as well, and a correct analysis should include chronological age as a covariate, as in the Alzheimer disease example that we report.

A preprint which appeared while this paper was under review [45] suggests that age acceleration may result from confounding of age with other phenomena such as blood cell composition, and indicates that adding further age-predicting loci to a clock model reduces association of mortality with age acceleration.

The clock model has interesting and useful characteristics, but it is an extremely narrow summary of the DNA methylation profile based on only 353 CpG sites representing 1.15 × 10^−5^% of the methylome. EWAS, association tests of the full DNA methylation profile, using appropriate genome-wide confidence limits are much more likely to lead to biological insights.

## Conclusion

The age prediction properties of both Horvath [8] and Hannum et al. [6] DNA methylation clock models begin to degrade as subjects enter old age. This is at least partly due to saturation, i.e., DNA methylation proportion at some loci approaching 0 or 1, and confounding with the effects of other age-related processes will also play a role. It is likely that this could be ameliorated with additional loci and/or further refined modeling of the currently used set. Association tests using age acceleration should incorporate age as a covariate (as should those using DNA methylation values for individual loci) to avoid spurious associations.

## Methods

This study was designed to investigate age prediction from DNA methylation profiles across multiple brain regions and blood, especially in older subjects. It uses a number of existing data sets.

### Samples

#### Tissue samples

Brain tissue samples (London cohort) were obtained from individuals diagnosed with Alzheimer’s disease (AD, *n* = 61) and from non-demented elderly control individuals (CON, *n* = 31) through the MRC London Neurodegenerative Disease Brain Bank as described in Lunnon et al. [9, 46]. In total, four brain regions were analyzed (prefrontal cortex (PFC), the entorhinal cortex (EC), the superior temporal gyrus (STG), and the cerebellum (CER)) and pre-mortem blood from a subset of individuals, collected as part of the Biomarkers of AD Neurodegeneration study. A second independent cohort (Mount Sinai cohort) was obtained from the Mount Sinai Alzheimer’s disease and Schizophrenia Brain Bank. This cohort consisted of two brain regions (PFC and STG) for 75 AD and 72 CON donors [12, 13].

#### Population sample: the UK Household Longitudinal Study (UKHLS)

UKHLS is an annual household-based panel study which started collecting information about the social, economic, and health status of its participants in 2009. Our analysis data set is drawn from one of the arms of UKHLS, namely, the British Household Panel Survey (BHPS), which merged with UKHLS in 2010 at the start of wave two. UKHLS collected additional health information, including blood samples for genetic and epigenetic analysis, at wave 3 for BHPS (www.understandingsociety.ac.uk). DNA methylation profiling and initial analysis are described in [10, 47].

#### Methylomic profiling

DNA from the London cohort tissue samples were bisulfite-treated using Zymo EZ 96 DNA methylation kit (Zymo Research) according to the manufacturer’s protocol. DNA methylation levels were assessed on an Illumina HiScan System using the Illumina Infinium HumanMethylation450 BeadChip as previously described by Lunnon et al. [9]. Raw signal intensities and probes for the London cohort were extracted using Illumina Genome Studio software and were transformed into beta values using the Bioconductor wateRmelon package [48]. These were later normalized using the method implemented in the Horvath [8] script. Data is available from both cohorts under GEO accession numbers GSE59685 (London cohort) and GSE80970 (Mount Sinai cohort).

One thousand one hundred ninety-three DNA samples from UKHLS were bisulfite-treated using Zymo EZ 96 DNA methylation kit (Zymo Research) according to the manufacturer’s protocol. DNA methylation levels were assessed on an Illumina HiScan System (Illumina) using the Illumina Infinium Epic Methylation BeadChip, and samples were randomly assigned to chips and plates to minimize batch effects. Furthermore, in order to resolve any experimental inconsistencies, and to approve data quality, a fully methylated control (CpG Methylated HeLa Genomic DNA; New England BioLabs, MA, USA) was included in a random position on each plate. Raw signal intensities and probes for UKHLS were extracted using Illumina Genome Studio software and were transformed into beta values using the Bioconductor bigmelon package (10.18129/B9.bioc.bigmelon) [49]. These were later normalized using dasen function from the wateRmelon package [48]. After QC, a final *n* of 1175 was reached.

#### DNA methylation age prediction

DNA methylation (DNAm) age was assessed for all samples of the London and Mt Sinai datasets on the R statistical environment (R Development Core Team, 2015) using the script provided by Horvath [8] as well as through the online DNAm Age Calculator (https://dnamage.genetics.ucla.edu/). These methods predicted age based on the DNAm coefficients of 353 CpG sites. The model (although not the custom normalization method) is also implemented in the agep() function of the wateRmelon package (version 1.17.0). This is expected to perform very similarly to the original Horvath protocol as long as reasonable preprocessing steps are used. A BA plot demonstrates that this is the case for the AD study samples (Additional file 1: Figure S1). The differences are small (sd of difference 1.8 years) and approximately normally distributed. The agep() function was used to predict the ages of the UKHLS samples for this study.

To maximize the number of brain samples included in our assessment of age prediction, publicly available 450KMethylation brain tissue datasets obtained from GEO (GSE40360, GSE53162, GSE59457, GSE61380, GSE61431, GSE67748, GSE67749, and GSE89702 [50–60]) along with the London and Mount Sinai cohorts were analyzed (Additional file 1: Table S1).

## Supplementary information

Additional file 1: : **Fig. S1.** Describing the differences between DNA methylation ages estimated with the Horvath 2013 calculator and the agep() function. As well as **Table S1.** Detailing additional data sets used in this study.

## Data Availability

The data used in this publication is all previously published. UKHLS DNA methylation data is available from the European Genome-phenome Archive under accession EGAS00001002836 (https://www.ebi.ac.uk/ega/home) [61]. Specific details can be found here (https://www.understandingsociety.ac.uk/about/health/data). Phenotypes linked to DNA methylation data are available through application to the METADAC (www.metadac.ac.uk). Publicly available 450KMethylation brain tissue datasets were obtained from GEO (GSE40360, GSE53162, GSE59457, GSE61380, GSE61431, GSE67748, GSE67749, and GSE89702 [50–60]). Detailed description is available from GEO and listed in Additional file 1: Table S1.
