# Supplementary material for: Systematic underestimation of the epigenetic clock and age acceleration in older subjects
Source: Genome Biol. 2019 Dec 17;20:283. doi: 10.1186/s13059-019-1810-4 (PMC6915902; doi:10.1186/s13059-019-1810-4)
Supplement: Supplementary file 1 — : Fig. S1. Describing the differences between DNA methylation ages estimated with the Horvath 2013 calculator and the agep() function. As well as Table S1. Detailing additional data sets used in this study. [file 13059_2019_1810_MOESM1_ESM.docx]

Figure S1. Bland-Altman plot comparing age estimates in years between the Horvath (2013) age calculator and the agep() function from the wateRmelon package, using the AD study samples (Lunnon et al 2014, GSE59685).

**Table S1** Breakdown of the phenotypes, tissues, and ages of the additional brain datasets

| GEO  Accession | Phenotype | Tissue | N | Age  Range | Age  (mean ± SD) | Reference |
| --- | --- | --- | --- | --- | --- | --- |
| GSE40360 | Multiple Sclerosis | White matter | 27 cases  19 control | 35 – 78  53 – 81 | 55.33 ± 9.99  66.32 ± 8.50 | Huynh *et al*., 2014 |
| GSE53162 | Autism | Temporal cortex | 6 cases  10 controls | 2 – 51  22 – 56 | 21.83 ± 17.42  39.6 ± 13.06 | Ladd-Acosta *et al*., 2014 |
|  |  | Prefrontal cortex | 6 cases  5 controls | 2 – 29  19 – 56 | 17.5 ± 10.82  34.8 ± 15.02 |  |
|  |  | Cerebellum | 7 cases  6 controls | 4 – 39  2 – 26 | 14.00 ± 12.34  16.67 ± 10.03 |  |
| GSE59457 | HIV | Cerebellum | 20 cases  4 controls | 26 – 64  38 – 63 | 43.79 ± 9.49  51.75 ± 10.31 | Horvath and Levine, 2015 |
|  |  | Frontal lobe | 2 cases  4 controls | 44 – 48  38 – 63 | 46.35 ± 2.48  51.75 ± 10.31 |  |
|  |  | Hippocampus | 4 controls | 38 – 63 | 51.75 ± 10.31 |  |
|  |  | Medial frontal cortex | 18 cases | 26 – 64 | 44.34 ± 10.57 |  |
|  |  | Midbrain | 2 controls | 38 – 53 | 45.5 ± 10.61 |  |
|  |  | Occipital cortex | 59 cases  13 controls | 26 – 68  32 – 64 | 49.89 ± 9.23  49.69 ± 11.57 |  |
|  |  | Temporal cortex | 4 controls | 38 – 63 | 51.75 ± 10.31 |  |
| GSE67748 | HIV | Cerebellum | 8 cases  12 controls | 27 – 64  15 – 85 | 44.00 ± 13.56  66.67 ± 22.73 | Horvath and Levine,2015 |
| GSE67749 | HIV | Frontal lobe | 8 cases  25 controls | 27 – 64  15 – 95 | 44.00 ± 13.56  72.72 ± 17.80 | Horvath and Levine, 2015 |
| GSE61380 | Schizophrenia | Prefrontal cortex | 18 cases  15 controls | 24 – 73  21 – 69 | 45.50 ± 16.61  42.20 ± 14.85 | Pidsley *et al*., 2014 |
| GSE61431 | Schizophrenia | Cerebellum | 21 cases  23 controls | 31 – 87  25 – 96 | 61.76 ± 16.61  61.39 ± 17.15 | Pidsley *et al*., 2014 |
|  |  | Frontal Cortex | 20 cases  23 controls | 32 – 87  25 – 96 | 62.05 ± 14.81  62.04 ± 17.09 |  |
| GSE89702 | Schizophrenia | Striatum | 37 cases  45 controls | 24 – 87  21 – 96 | 55.11 ± 17.58  56.67 ± 20.08 | Viana *et al*., 2016 |
|  |  | Hippocampus | 14 cases  13 controls | 31 – 79  25 – 95 | 59.71 ± 13.59  64.46 ± 18.90 |  |
|  |  | Cerebellum | 16 cases  17 controls | 24 – 70  21 – 72 | 45.69 ± 16.93  44.59 ± 15.79 |  |
